# A Multimodal Approach to Addictive-Like Behavior in Stock Trading: A Case Report

**DOI:** 10.7759/cureus.101490

**Published:** 2026-01-13

**Authors:** Subramanian Saradha, Rajesh Kumar, Manoj K Sharma

**Affiliations:** 1 Clinical Psychology, National Institute of Mental Health and Neurosciences (NIMHANS), Bengaluru, IND

**Keywords:** behavioral addiction, case report, f&o trading, multimodal intervention, shut clinic, stock trading addiction

## Abstract

Stock trading has become increasingly popular due to the advent of mobile trading apps and internet platforms, which provide individuals with instant access to financial markets. While these tools have made trading faster and more convenient, excessive or problematic trading can lead to financial loss, psychological distress, and significant dysfunction in various areas of life. Recent trends suggest that problematic stock trading is emerging as a mental health concern, with individuals increasingly seeking treatment.

We present the case of a 29-year-old employed male exhibiting addictive-like behavior related to stock trading. He reported a loss of control over trading, persistent preoccupation with market activity, compulsive chasing of financial losses, and sleep disturbances. These issues resulted in significant impairment in personal, social, and financial domains. The Stock Addiction Inventory (SAI) was used to assess the severity of his stock trading behavior, indicating a severe addiction.

A multimodal intervention approach was implemented to manage his condition. Pre-post assessments indicated improvements in the severity of stock trading behavior and psychological well-being. This case study underscores the importance of testing multimodal interventions for the treatment of addictive-like stock trading behavior on a larger scale. Early recognition and tailored treatment approaches could be essential in addressing this emerging behavioral problem.

## Introduction

Stock trading is the activity of buying and selling shares of publicly listed companies to earn profit from changes in their market prices. Stock trading includes the purchase and sale of equity or stocks, bonds, futures and options (F&O), commodities, currencies, and derivatives [1]. In India, stock trading has grown substantially due to the increased accessibility of online platforms and mobile-based trading apps. Retail investor participation has surged, as reflected in the unprecedented rise in demat accounts from 36 million in 2019 to 194 million in the financial year 2025 (FY25) [2]. Recent data from both depositories - the Central Depository Services Limited (CDSL) and the National Securities Depository Limited (NSDL) - show that the number of demat accounts in India has surpassed 200 million. A majority of account holders are young individuals under the age of 30, with approximately 75% of new accounts opened by young retail investors [3].

Short-term trading activities that are focused solely on profit-making are considered high risk. Furthermore, the use of high leverage (borrowed funds), margin trading, and pledging stocks to obtain funds from brokers in order to trade larger amounts may further increase financial risk and potential losses. The Securities and Exchange Board of India (SEBI) reported that over 93% of retail traders incurred losses in F&O trades over the last three to four years [4]. Moreover, 70% of intraday traders experienced losses in FY23 [5]. Trading may develop into an addictive-like behavior, sharing many phenomenological similarities with gambling disorders. In particular, intraday and F&O trading mirror gambling behaviors. Grall-Bronnec et al. reported that individuals with excessive trading exhibited addictive-like traits such as preoccupation with trading; deception to family or others to conceal trading involvement, repeated failed attempts to control or stop trading; and chasing losses by returning to trade in hopes of recovering money [6]. A more recent model (The Griffith’s component model) describes the criteria for addictive like behaviors are salience, mood modifications, tolerance, withdrawal, conflict, and relapse [7]. The International Classification of Diseases (ICD-11) recognizes gambling and gaming disorders as behavioral addictions. Other problematic behaviors, such as stock trading addiction, may fall under “other specified disorders due to addictive behaviors” [8,9]. Stock trading addiction is characterized by persistent, recurrent problematic trading that interferes with personal, familial, and professional functioning. It includes a gradual loss of control over trading, as well as tolerance and withdrawal symptoms similar to substance use disorders [1]. These characteristics have been observed amongst a specific group of stock trading investors who engage in problematic stock trading [10]. The problematic trading or addictive-like trading has been an area of concern in view of easy availability through online investing apps and platforms, options of pay later, borrowing money, or pledging options.

## Case presentation

We report the case of Mr. D., a 29-year-old male from an upper-middle socioeconomic background in India who sought consultation at the Services for Healthy Use of Technology (SHUT) Clinic. Mr. D. began stock trading four years ago via a mobile platform as a secondary source of income. Self-taught through independent research and reading, he engaged primarily in options, futures, and intraday trading. Initially, he invested small amounts and experienced profits for about a year. Over time, he became preoccupied with trading, constantly monitoring the market and frequently thinking about strategies to make profits. He reported experiencing anxiety during trading, along with stress both before and after trading hours. Significant losses brought about frustration, shame, guilt, low mood, feelings of worthlessness and hopelessness, as well as disrupted sleep patterns. As his trading volume increased substantially, so did his financial losses. Despite wanting to stop, he reported being unable to do so, indicating a loss of control. To continue trading and recover losses, he borrowed money from loan apps. He described a compulsive urge to trade, leading to social withdrawal and deception of family members regarding his behavior until the debt became unmanageable. He endorsed maladaptive cognitions such as “I can recover my losses in the next trade” and exhibited overconfidence bias, reinforcing the addictive cycle. His behavior resulted in a cumulative debt of ₹8 million (80 lakhs).

This case aligns with both the component model of behavioral addiction [7] as well as the ICD-11 criterion for behavioral addiction. These include salience, which refers to trading becoming central to his life; mood modification, indicating that trading influenced his emotional state; increasing frequency and investment amounts (tolerance); emotional distress when not trading (withdrawal); conflict with interpersonal or intrapsychic issues; and relapse, indicated by repeated returns to trading after attempts to stop.

Assessment

**Figure 1:**
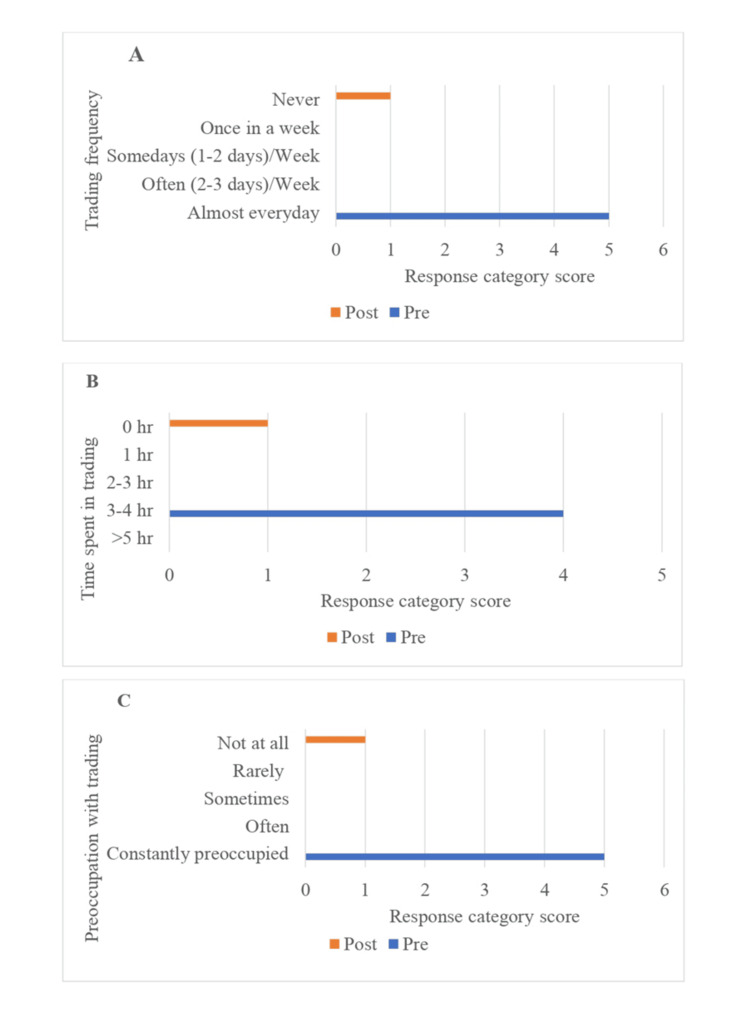
Changes in trading behavior from pre- to post-intervention: (A) frequency of trading, (B) average daily time spent trading, and (C) cognitive preoccupation with trading.

The Stock Addiction Inventory (SAI) [11], a nine-item tool rated on a 4-point Likert scale (0 = Never to 3 = Almost Always), was used to assess severity. Items include "borrowed money or sold items for trading," "felt guilty about excessive trading," and "trading caused financial problems." The SAI also has items assessing core addictive behavior characteristics such as "Felt a strong urge to make money from trading" and "Spent increasing time/money on trading." Scores range from 0 to 27. Mr. D. scored 24, indicating severe stock trading addiction. A Visual Analogue Scale (VAS) was also used pre- and post-intervention to assess trading frequency, time spent, and preoccupation (Figure 1). He reported spending ₹500,000 in a single trading day and incurring a total debt of ₹8,000,000 (Rupees 80 lakhs) due to trading.

Multimodal behavioral intervention

A multimodal behavioral intervention previously shown to be effective for Internet Gaming Disorder [12] was adapted for addictive-like behaviors related to stock trading. The intervention program consisted of 10 sessions, each lasting 60 minutes. The first four sessions were weekly, followed by six bi-weekly sessions. Intervention components included psychoeducation about addiction and trading behavior; digital fasting and stimulus control (e.g., staying away from trading apps); delegating financial control to family; mindfulness-based relaxation for managing anxiety; stress inoculation techniques; cognitive restructuring and journaling for maladaptive beliefs (e.g., chasing losses); The four D’s technique: Deep breathing, Drinking water, Discussion with family, and Decreasing mobile use to him as relapse prevention techniques. The patient reported that these techniques were effective in reducing his trading behavior.

## Discussion

Following the multimodal behavioral intervention, the patient demonstrated a marked reduction in trading-related behaviors, as evidenced by decreases in trading frequency, time spent on trading, and cognitive preoccupation with trading (Figure 1). These behavioral improvements were accompanied by a substantial reduction in overall severity, with the SAI score decreasing from 24 at pre-intervention to 4 at post-intervention, indicating clinically meaningful improvement in loss of control and cognitive salience associated with problematic trading. These gains were maintained at the three-month follow-up.

Financial trading is traditionally viewed as distinct from gambling and is commonly framed as an investment strategy. However, recent developments, such as the proliferation of online and mobile trading platforms and the rise of high-risk trading practices (e.g., day trading, frequent trading, high trading volume, and margin trading), have increasingly blurred the boundary between trading and gambling [13]. Studies have reported a gambling tendency in stock trading, particularly in those who are involved in a higher frequency of stock trading, day trading, and involved in derivatives trading [14]. Similarly, it has been reported that speculation and gambling have a strong relationship, which is indicative of a form of behavioral addiction [10], and F&O trading inherently involves speculation.

The present case demonstrated many characteristics similar to addictive like behavior and gambling characteristics, such as compulsive daily trading, preoccupation, tolerance (need to trade more), difficulty in cutting down trading, chasing losses, deception, anxiety, guilt, and reliance on others for financial support. His SAI score indicated severe stock trading addiction, validating his problem severity or diagnosis. Online investment platforms and apps provide easy accessibility to users, and they may engage in trading at workplaces as a secondary income generation, and slowly turn into spending more time in trading, more frequent logging, etc., engaging in frequent and high numbers of trades. Young and novice traders can be more prone to addictive or problematic trading because they are more likely to have stimulation-seeking motives and overconfidence bias [15]. This case report highlights the potential utility of a multimodal intervention in managing emerging behavioral addictions such as problematic stock trading. However, findings should be interpreted with caution, as evidence from larger, well-designed studies is warranted beyond single-case observations. SEBI’s recent reports emphasize the need to better understand retail traders' behavior, especially those involved in F&O and intraday trading. Future research should examine behavioral, personality, and neurocognitive characteristics of traders to improve the understanding of vulnerability factors associated with problematic stock trading. Although concerns regarding excessive and poorly regulated trading behaviors are increasing, clinically grounded empirical evidence remains sparse. Subsequent studies should incorporate objective assessments, appropriate inferential analyses, and longitudinal follow-up to strengthen methodological rigor and reproducibility, particularly in the evaluation of multimodal interventions. In parallel, there is a pressing need to develop preventive approaches, targeted interventions, and public health guidelines that promote responsible and regulated stock trading.

## Conclusions

This case demonstrates that problematic stock trading can manifest with core features of behavioral addiction, including impaired control, compulsive engagement, chasing losses, emotional distress, and functional impairment. Following intervention, the patient showed marked clinical improvement, evidenced by substantial reductions in SAI scores, trading frequency, time spent trading, and cognitive preoccupation. High-risk trading, particularly in F&O, appeared to function in a gambling-like manner, facilitated by easy access to online platforms and continuous market feedback. The findings suggest that structured multimodal interventions may be beneficial, while underscoring the need for systematic assessment and larger studies to clarify mechanisms and inform prevention and treatment approaches.
